# Finite Element Solution of Unsteady Mixed Convection Flow of Micropolar Fluid over a Porous Shrinking Sheet

**DOI:** 10.1155/2014/362351

**Published:** 2014-02-04

**Authors:** Diksha Gupta, Lokendra Kumar, Bani Singh

**Affiliations:** Department of Mathematics, Jaypee Institute of Information Technology, A-10, Sector 62, Noida, Uttar Pradesh 201307, India

## Abstract

The objective of this investigation is to analyze the effect of unsteadiness on the mixed convection boundary layer flow of micropolar fluid over a permeable shrinking sheet in the presence of viscous dissipation. At the sheet a variable distribution of suction is assumed. The unsteadiness in the flow and temperature fields is caused by the time dependence of the shrinking velocity and surface temperature. With the aid of similarity transformations, the governing partial differential equations are transformed into a set of nonlinear ordinary differential equations, which are solved numerically, using variational finite element method. The influence of important physical parameters, namely, suction parameter, unsteadiness parameter, buoyancy parameter and Eckert number on the velocity, microrotation, and temperature functions is investigated and analyzed with the help of their graphical representations. Additionally skin friction and the rate of heat transfer have also been computed. Under special conditions, an exact solution for the flow velocity is compared with the numerical results obtained by finite element method. An excellent agreement is observed for the two sets of solutions. Furthermore, to verify the convergence of numerical results, calculations are conducted with increasing number of elements.

## 1. Introduction

In the last few decades, the interest for non-Newtonian fluids has considerably increased due to their connection with applied sciences. The motion of these fluids plays essential role not only in theory but also in many industrial processes. Among the various non-Newtonian fluid models, the micropolar fluids have acquired the special attention in recent years due to their applications in polymeric fabrication, materials processing, and biotechnology. Flow and heat transfer behaviour of these fluids cannot be described by the classical theory of continuum mechanics. Eringen [1] has formulated the theory of micropolar fluids which describes the physics of such fluids. In the micropolar fluid theory, two new variables to the velocity are added which were not presented in the Navier-Stokes model. These variables are microrotations that represent spin and microinertia tensors which describe the distribution of atoms and molecules inside the microscopic fluid particles. This class of fluids represents, mathematically, many industrial important fluids such as paints, lubricants, polymers, human and animal blood, colloidal suspensions, and liquid crystals. The theory and applications of micropolar fluids can be found in the books by Eringen [2] and Bég et al. [3]. Later Eringen [4] extended the theory of micropolar fluids to thermo-microfluids. This theory takes into account thermal effects, that is, heat conduction, convection, and dissipation. These effects were not included in the classical field theories.

The boundary layer flow induced by stretching surface is of great practical interest because it occurs in a number of engineering processes. Boundary layer flow of a Newtonian fluid caused by a linearly stretching sheet was first examined by Crane [5]. He gave a similarity solution in closed analytical form for the steady two-dimensional problem. The flow and heat transfer of micropolar fluid past a continuously moving porous plate was analyzed by Takhar and Soundalgekar [6]. Hassanien and Gorla [7] conducted numerical computations to examine the heat transfer characteristics of micropolar fluid past a nonisothermal sheet with suction and blowing. Thereafter, various aspects of micropolar fluid flows from a stretching surface have been reported by El-Arabawy [8], Nazar et al. [9], Kumar [10], Ishak [11], and Rawat et al. [12].

The above-mentioned studies deal with a steady flow only. However, in certain cases, the flow and heat transfer can be unsteady due to a sudden stretching of the flat sheet or by a change of the temperature or heat flux of the sheet. Devi et al. [13] investigated the unsteady three-dimensional flow caused by a stretching flat surface. Using the finite difference scheme in combination with the quasi-linearization technique Rajeswari, and Nath [14] reported on the unsteady flow over a stretching surface in a rotating fluid. Exact similarity solution of the heat transfer in a liquid film on an unsteady stretching surface was obtained by Andersson et al. [15]. Abd El-Aziz [16] studied numerically the effect of radiation on the heat and fluid flow over an unsteady stretching sheet showing that the heat transfer rate is an increasing function of radiation and unsteadiness parameters. Lok et al. [17] obtained the numerical solution for the unsteady boundary layer flow of a micropolar fluid near the stagnation point of a plane surface. By taking into account strong and weak concentration of microelements, Hayat et al. [18] examined the effect of magnetic field on the time-dependent flow of micropolar fluid between stretching sheets. Bachok et al. [19] studied theoretically the unsteady boundary layer flow and heat transfer due to a stretching sheet, showing that the surface shear stress and the heat transfer rate are an increasing function of unsteadiness parameter.

Recently, the boundary layer flow of incompressible fluid over a shrinking sheet has attracted extensive attention due to its increasing applications in polymeric materials processing. This type of flow was first examined analytically by Wang [20]. Later, Miklavčič and Wang [21] proved the existence and uniqueness of the solution for the flow over a shrinking sheet. From the physical point of view, steady flow over a shrinking sheet is not possible since the generated vorticity is not confined within the boundary layer. To overcome this difficulty, the flow requires a certain amount of external opposite force at the sheet. This has been extensively discussed in the literature. Some important references are Miklavčič and Wang [21], Wang [22], and Bachok et al. [23]. Closed form exact solutions of MHD Newtonian flow over a shrinking sheet were derived by Fang and Zhang [24]. Using shooting method, Bhattacharyya and Layek [25] obtained the numerical solutions for the flow and heat transfer over a porous shrinking sheet in the presence of thermal radiation. The shrinking sheet problem was extended to micropolar fluids by Ishak et al. [26] who considered stagnation point flow. Yacob and Ishak [27] analyzed the flow and heat transfer over a shrinking sheet immersed in a micropolar fluid. Recently, numerical simulation of mixed convection flow of micropolar fluid over a shrinking sheet with thermal radiation was conducted by Gupta et al. [28].

Many researchers have investigated the unsteady flow over a shrinking sheet. Fang et al. [29] examined the unsteady viscous flow over a shrinking surface with mass suction and highlighted the deviation in flow behaviour for an unsteady shrinking sheet compared with an unsteady stretching sheet. Effect of radiation on the unsteady flow and heat transfer induced by a permeable shrinking sheet was reported by Ali et al. [30]. Bhattacharyya [31] examined the effects of radiation and heat source/sink on unsteady flow and heat transfer past a shrinking sheet with suction/injection. Numerical solution for the unsteady stagnation point flow and heat transfer over a stretching/shrinking sheet with prescribed heat flux was analyzed by Suali et al. [32]. They found that the skin friction and local Nusselt number increase with unsteadiness. Mahapatra and Nandy [33] studied the unsteady stagnation-point flow and heat transfer over a linearly shrinking sheet in the presence of velocity and thermal slips.

In many of the studies described above, buoyancy effect plays a significant role. The buoyancy force, developed from the temperature difference, induces a longitudinal pressure gradient which in turn modifies the flow field and the rate of heat transfer from the surface. A computational study of unsteady mixed convection flow in stagnation region adjacent to a vertical surface was conducted by Devi et al. [34]. Ishak et al. [35] presented the numerical solution for the unsteady mixed convection flow and heat transfer over a stretching vertical sheet. Both assisting and opposing flow cases were taken into consideration. Unsteady mixed convection boundary layer flow over a stretching vertical surface in the presence of velocity slip was presented by Mukopadhyay [36]. Sharma et al. [37] employed element-free Galerkin method to study the mixed convection flow and heat transfer of a viscous fluid over an unsteady stretching sheet in a porous medium. Using Keller-Box method, Vajravelu et al. [38] examined the effects of variable thermal conductivity, thermal radiation and the thermal buoyancy on the unsteady fluid flow and heat transfer at a porous stretching sheet.

The present paper investigates the unsteady mixed convection flow and heat transfer of an incompressible micropolar fluid over a vertical shrinking sheet with time-dependent suction at the sheet. Viscous dissipation effects are also included in the energy equation. The velocity and temperature of the sheet are assumed to vary with the horizontal coordinate *x*  and time *t*. Similarity transformations are employed for the conversion of governing time-dependent boundary layer equations into ordinary differential equations. These equations are then solved numerically using finite element method. The influence of suction parameter, unsteadiness parameter, buoyancy parameter, and Eckert number has been depicted graphically. The skin friction and the rate of heat transfer have also been computed and tabulated for these parameters. The current study has applications in industrial polymeric materials processing and has not been considered so far to the knowledge of the authors.

## 2. Mathematical Model

**Figure 1:**
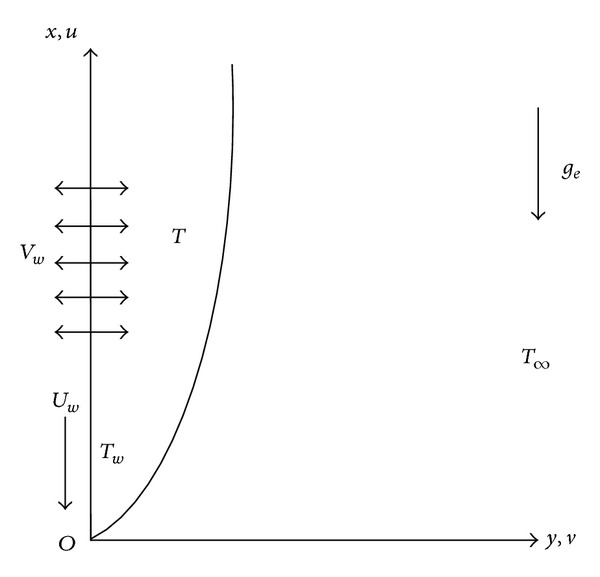
Physical model and coordinate system.

Consider the unsteady, mixed convection, and boundary layer flow of an incompressible micropolar fluid past a permeable shrinking sheet. A schematic representation of the physical model and coordinate system is depicted in Figure 1. It is assumed that, for time *t* < 0, the fluid and heat flows are steady. The unsteady fluid and heat flow start at *t* = 0. The velocity of shrinking sheet is *U*_*w*_(*x*, *t*) = −*ax*/(1 − *et*) and the temperature of the sheet is *T*_*w*_(*x*, *t*) = *T*_*∞*_ + *bx*/(1 − *et*), where *a*, *e* are constants (with *a* > 0, *e* ≥ 0, wher *et* < 1) and both have dimension (time)^−1^, while *b* is a constant and has dimensions (temperature/length). The coordinate system is such that *x*-axis is taken along the shrinking sheet in a direction opposite to sheet motion and *y*-axis is normal to it. Time-dependent suction is considered normal to the shrinking sheet. The physical properties of the fluid are assumed to be constant except density variation due to temperature difference which is used only to express the body force term as the buoyancy term. The effect of viscous dissipation is also included in the energy equation. Under these assumptions the governing boundary layer equations for unsteady flow over a shrinking sheet may be presented as follows.

 Continuity equation
  (1) $$\begin{array}{c} \frac{\partial u}{\partial x}+\frac{\partial v}{\partial y}=0. \end{array}$$
 Momentum equation
  (2) $$\begin{array}{c} \frac{\partial u}{\partial t}+u\frac{\partial u}{\partial x}+v\frac{\partial u}{\partial y}=\frac{\left(\mu +S\right)}{\rho }\frac{\partial^{2}u}{\partial y^{2}}+\frac{S}{\rho }\frac{\partial N}{\partial y}+g_{e}\beta \left(T-T_{\infty }\right). \end{array}$$
 Angular momentum equation
  (3) $$\begin{array}{c} \left(\frac{\partial N}{\partial t}+u\frac{\partial N}{\partial x}+v\frac{\partial N}{\partial y}\right)=\frac{\gamma }{\rho j}\frac{\partial^{2}N}{\partial y^{2}}-\frac{S}{\rho j}\left(2N+\frac{\partial u}{\partial y}\right). \end{array}$$
 Energy equation
  (4) $$\begin{array}{c} \frac{\partial T}{\partial t}+u\frac{\partial T}{\partial x}+v\frac{\partial T}{\partial y}=\frac{\kappa }{\rho c_{p}}\frac{\partial^{2}T}{\partial y^{2}}+\frac{\left(\mu +S\right)}{\rho c_{p}}{\left(\frac{\partial u}{\partial y}\right)}^{2}. \end{array}$$

The associated boundary conditions are

(5) y=0:u=Uw(x,t),  v=Vw,N=−12∂u∂y,  T=Tw(x,t),y⟶∞:u⟶0, N⟶0, T⟶T∞.

It is assumed that *V*_*w*_ is a variable distribution of suction through porous sheet and is given by $V_{w}=(1/\sqrt{1-et})v_{0}$ (Bhattacharyya et al. [39]).

Introducing the similarity variable *η* and the dimensionless functions *f*, *g*, and *θ* as follows:

(6) η=(aν(1−et))1/2y,  ϕ=(aν(1−et))1/2xf,N=(a(1−et))3/2xνg,  θ=T−T∞Tw−T∞,

where *ϕ* is a stream function defined as *u* = ∂*ϕ*/∂*y*, *v* = −∂*ϕ*/∂*x* which identically satisfies the continuity equation (1). On applying the transformations, (2)–(4) are reduced to

(7) (1+K)f′′′+ff′′−(f′)2+Kg′  +σθ−τ(f′+12ηf′′)=0,(1+K2)g′′+fg′−f′g−K(2g+f′′)  −τ(32g+12ηg′)=0,θ′′+Pr(fθ′−f′θ)+(1+K)Pr⁡Ec(f′′)2  −Pr⁡τ(θ+12ηθ′)=0,

and the corresponding boundary conditions (5) now transform to

(8) $$\begin{array}{c} f=-\lambda ,\;f^{\prime }=-1,\;g=-\frac{1}{2}f^{\prime \prime },\;\theta =1\;\text{at}\;\;\eta =0,\\ f^{\prime }=0,\;g=0,\;\theta =0\;\text{as}\;\;\eta ⟶\infty , \end{array}$$

where prime denotes the differentiation with respect to *η* only, *K* = *S*/*μ* (coupling constant parameter), *σ* = Gr_*x*_/(Re_*x*_)^2^ (buoyancy parameter), Gr_*x*_ = *g*_*e*_ *β*  (*T*_*w*_ − *T*_*∞*_)*x* ^3^/*ν* ^2^ (local Grashof number), *τ* = *a*/*e* (unsteadiness parameter), Pr = *μc*_*p*_/*κ* (Prandtl number), Ec = *U*_*w*_^2^/*c*_*p*_(*T*_*w*_ − *T*_*∞*_) (Eckert number), and $\lambda =v_{0}/\sqrt{a\nu }$ (suction parameter); *λ* > 0 corresponds to suction and *λ* < 0 corresponds to injection.

The quantities of physical interest, namely, the local skin friction coefficient and the rate of heat transfer, are, respectively, prescribed by

(9) $$\begin{array}{c} C_{f}=\frac{2\tau_{w}}{\rho U_{w}^{2}},\;\;\text{N}{\text{u}}_{x}=\frac{qx}{\kappa \left(T_{w}-T_{\infty }\right)}, \end{array}$$

where the local wall shear stress *τ*_*w*_ and the heat transfer from the sheet *q* are given by

(10) $$\begin{array}{c} \tau_{w}=-{\left[\left(\mu +S\right)\frac{\partial u}{\partial y}+SN\right]}_{y=0},\;\;q=-\kappa {\left(\frac{\partial T}{\partial y}\right)}_{y=0}. \end{array}$$

Using the similarity transformations given in (6), we obtain the following:

(11) $$\begin{array}{c} C_{f}{\left(\text{R}{\text{e}}_{x}\right)}^{1/2}=-\left(2+K\right)f^{\prime \prime }\left(0\right),\;\;\frac{\text{N}{\text{u}}_{x}}{{\left(\text{R}{\text{e}}_{x}\right)}^{1/2}}=-\theta^{\prime }\left(0\right), \end{array}$$

where Re_*x*_ = *U*_*w*_ *x*/*ν* is the local Reynolds number.

## 3. Method of Solution

The set of differential equations given in (7) are non-linear and therefore, cannot be solved analytically. Thus for the solution of this problem, finite element method has been implemented. Comprehensive details of this method can be found in Reddy [40]. In order to apply finite element method first we assume

(12) $$\begin{array}{c} f^{\prime }=h. \end{array}$$

Using (12), equation (7) reduces to

(13) (1+K)h′′+fh′−h2+Kg′+σθ−τ(h+12ηh′)=0,(1+K2)g′′+fg′−hg−K(2g+h′)  −τ(32g+12ηg′)=0,θ′′+Pr⁡(fθ′−hθ)+(1+K)Pr⁡Ec(h′)2  −Pr⁡τ(θ+12ηθ′)=0,

and the corresponding boundary conditions now become

(14) $$\begin{array}{c} f=-\lambda ,\;h=-1,\;g=-\frac{1}{2}h^{\prime },\;\theta =1\;\text{at}\;\;\eta =0,\\ h=0,\;g=0,\;\theta =0\;\text{as}\;\;\eta ⟶\infty . \end{array}$$

It has been observed that, for *η* > 8, there is no appreciable effect on the results. Therefore, for the computational purposes, *∞* can be fixed at 8.

### 3.1. Variational Formulation

The variational form associated with (12) and (13) over a typical two-noded linear element (*η*_*e*_, *η*_*e*+1_) is given by

(15) ∫ηeηe+1w1{f′−h}dη=0,∫ηeη  e+1  w2{(1+K)h′′+fh′−h2+Kg′+σθ−τ(h+12ηh′)}dη=0,∫ηeηe+1w3{(1+K2)g′′+fg′−hg−K(2g+h′)−τ(32g+12ηg′)}dη=0,∫ηeηe+1w4{θ′′+Pr⁡(fθ′−hθ)+(1+K)Pr⁡Ec(h′)2−Pr⁡τ(θ+12ηθ′)}dη=0,

where *w*_1_, *w*_2_, *w*_3_, and *w*_4_  are weight functions which may be viewed as the variation in *f*, *h*, *g*, and *θ*, respectively.

### 3.2. Finite Element Formulation

The finite element model can be obtained from (15) by substituting finite element approximations of the form

(16) f=∑j=12fjψj,  h=∑j=12hjψj,g=∑j=12gjψj,  θ=∑j=12θjψj,

with *w*_1_ = *w*_2_ = *w*_3_ = *w*_4_ = *ψ*_*i*_  (*i* = 1, 2), where *ψ*_*i*_  are the shape functions for a typical element (*η*_*e*_, *η*_*e*+1_) and are taken as follows:

(17) $$\begin{array}{c} \psi_{1}=\frac{\eta_{e+1}-\eta }{\eta_{e+1}-\eta_{e}},\;\;\psi_{2}=\frac{\eta -\eta_{e}}{\eta_{e+1}-\eta_{e}},\;\;\eta_{e}\leq \eta \leq \eta_{e+1}. \end{array}$$

The finite element model of the equations thus formed is given by

(18) $$\begin{array}{c} \left[\begin{array}{cccc} \left[K^{11}\right] & \left[K^{12}\right] & \left[K^{13}\right] & \left[K^{14}\right]\\ \left[K^{21}\right] & \left[K^{22}\right] & \left[K^{23}\right] & \left[K^{24}\right]\\ \left[K^{31}\right] & \left[K^{32}\right] & \left[K^{33}\right] & \left[K^{34}\right]\\ \left[K^{41}\right] & \left[K^{42}\right] & \left[K^{43}\right] & \left[K^{44}\right] \end{array}\right]\left[\begin{array}{c} \left\{f\right\}\\ \left\{h\right\}\\ \left\{g\right\}\\ \left\{\theta \right\} \end{array}\right]=\left[\begin{array}{c} \left\{b^{1}\right\}\\ \left\{b^{2}\right\}\\ \left\{b^{3}\right\}\\ \left\{b^{4}\right\} \end{array}\right], \end{array}$$

where [*K* ^*mn*^] and [*b* ^*m*^] (*m*, *n* = 1, 2, 3, 4) are the matrices of order 2 × 2, and 2 × 1 respectively and are defined as follows:

(19) Kij11=∫ηeηe+1ψidψjdηdη,  Kij12=−∫ηeηe+1ψiψjdη,Kij13=Kij14=0,  Kij21=0,Kij22=−(1+K)∫ηeηe+1dψidηdψjdηdη+∫ηeηe+1f−ψidψjdηdη−∫ηeηe+1h−ψiψjdη−τ∫ηeηe+1ψiψjdη−τ2∫ηeηe+1ηψidψjdηdη,Kij23=K∫ηeηe+1ψidψjdηdη,  Kij24=σ∫ηeηe+1ψiψjdη,Kij31=0,  Kij32=−K∫ηeηe+1ψidψjdηdη,Kij33=−(1+K2)∫ηeηe+1dψidηdψjdηdη+∫ηeηe+1f−ψidψjdηdη−∫ηeηe+1h−ψiψjdη−2K∫ηeηe+1ψiψjdη−32τ∫ηeηe+1ψiψjdη−τ2∫ηeηe+1ηψidψjdηdη,Kij34=0,  Kij41=0,Kij42=(1+K)Pr⁡Ec∫ηeηe+1ψidh−dηdψjdηdη,  Kij43=0,Kij44=−∫ηeηe+1dψidηdψjdηdη+Pr⁡∫ηeηe+1f−ψidψjdηdη−Pr⁡∫ηeηe+1h−ψiψjdη−Pr⁡τ∫ηeηe+1ψiψjdη−Pr⁡τ2∫ηeηe+1ηψidψjdηdη,bi1=0,  bi2=−(1+K)(ψidhdη)ηeηe+1,bi3=−(1+K2)(ψidgdη)ηeηe+1,  bi4=−(ψidθdη)ηeηe+1,

where $\overset{-}{f}\;=\sum_{i=1}^{2}{\overset{-}{f}}_{i}\psi_{i}$ and $\overset{-}{h}\;=\sum_{i=1}^{2}{\overset{-}{h}}_{i}\psi_{i}$ are assumed to be known. After the assembly of element equations, a system of non-linear equations is obtained; therefore, an iterative scheme must be utilized to solve it. The system is linearized by incorporating the functions $\overset{-}{f}\;$and $\overset{-}{h}\;$, which are assumed to be known at lower iteration level and the computations for*f*, *h*, *g*, and *θ* are then carried out for higher levels. This process is repeated until the desired accuracy of 0.00005 is attained. Convergence of the results with increasing number of elements is shown in Table 1. It is clear from the table that, for more than 160 elements no significant variation in the values of *f*, *h*, *g* and *θ* is observed. Thus the final results are reported for 160 elements. This confirms the mesh-independence of the present computation.

For steady state (*τ* = 0), viscous fluid (*K* = 0) and in the absence of buoyancy force (*σ* = 0), the exact solution for *f*(*η*), as obtained by Fang and Zhang [24] with *M* = 0, is given by *f*(*η*) = *λ* − (1 − *e* ^−*ηz*^)/*z*, where *z* = 0.5[*λ* + (*λ* ^2^ − 4)^1/2^].

The comparison of the flow velocity *f*′(*η*)  obtained by finite element method and the exact solution given by Fang and Zhang [24] is shown in Table 2. It is clear from the table that numerical results obtained are in complete agreement with the exact solution and thus confirm the validity and accuracy of the FEM.

## 4. Results and Discussion

**Figure 2:**
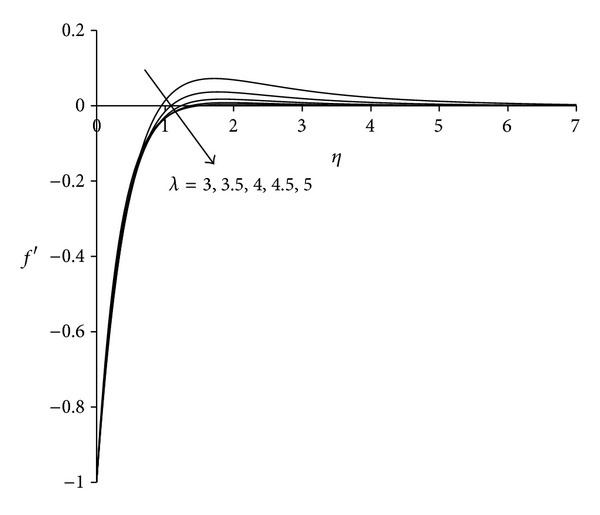
Velocity distribution for different *λ*  (*τ* = 1, *σ* = 3, Ec = 0.75).

**Figure 3:**
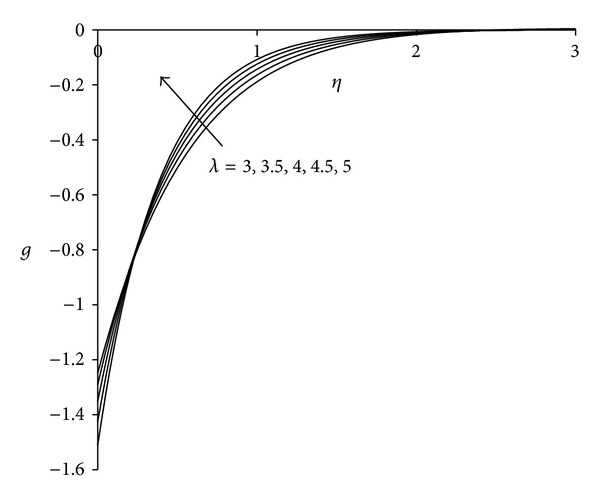
Microrotation distribution for different *λ*  (*τ* = 1, *σ* = 3, Ec = 0.75).

**Figure 4:**
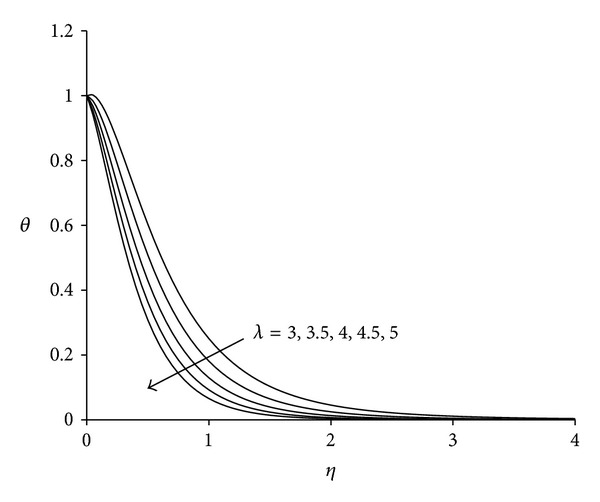
Temperature distribution for different *λ*  (*τ* = 1, *σ* = 3, Ec = 0.75).

**Figure 5:**
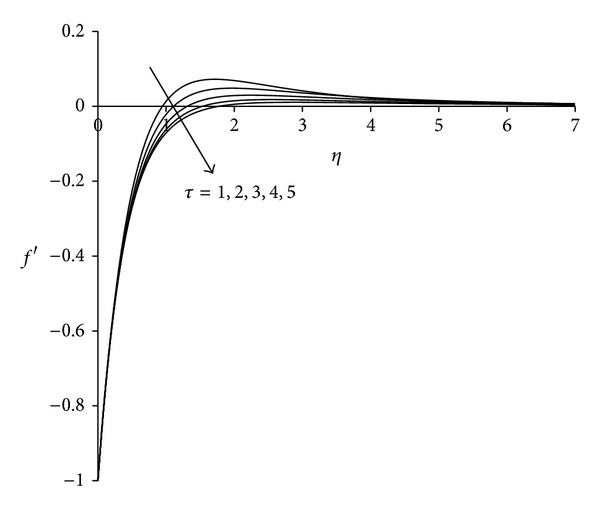
Velocity distribution for different *τ*  (*λ* = 3, *σ* = 3, Ec = 0.75).

**Figure 6:**
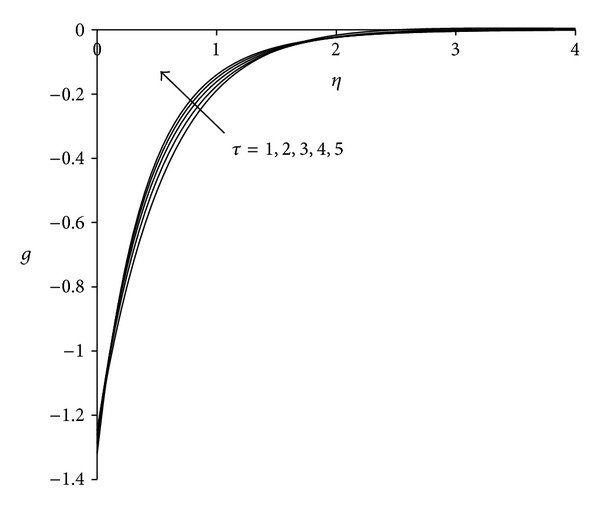
Microrotation distribution for different *τ*  (*λ* = 3, *σ* = 3, Ec = 0.75).

**Figure 7:**
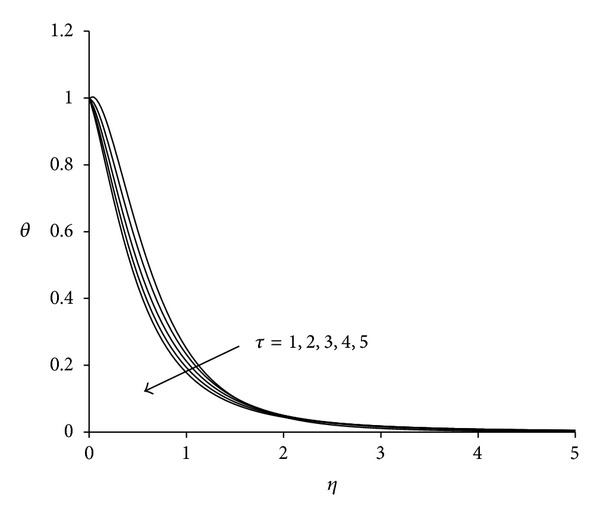
Temperature distribution for different *τ*  (*λ* = 3, *σ* = 3, Ec = 0.75).

**Figure 8:**
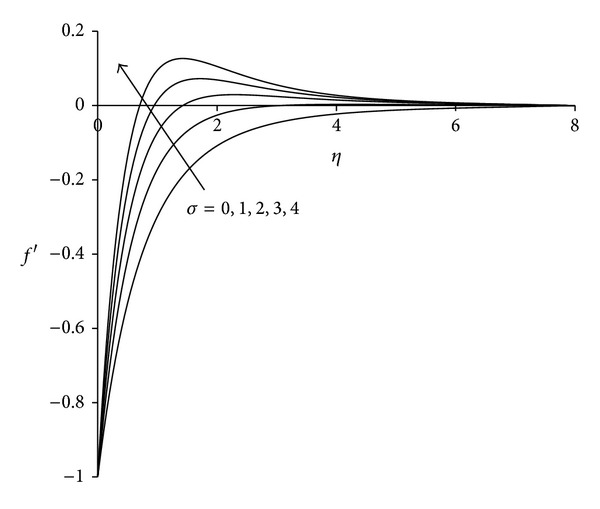
Velocity distribution for different *σ*  (*λ* = 3, *τ* = 1, Ec = 0.75).

**Figure 9:**
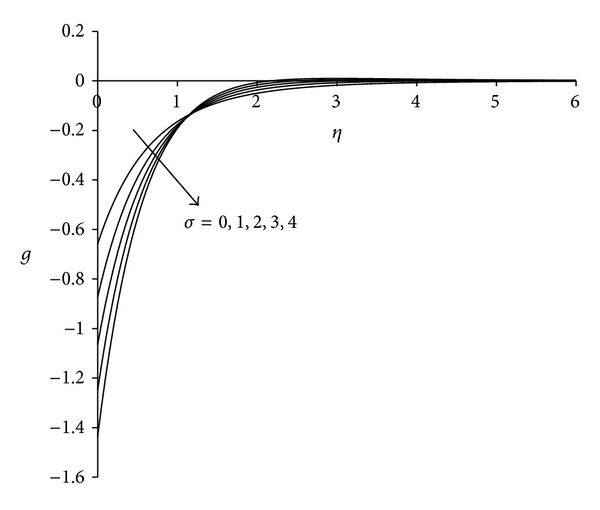
Microrotation distribution for different *σ*  (*λ* = 3, *τ* = 1, Ec = 0.75).

**Figure 10:**
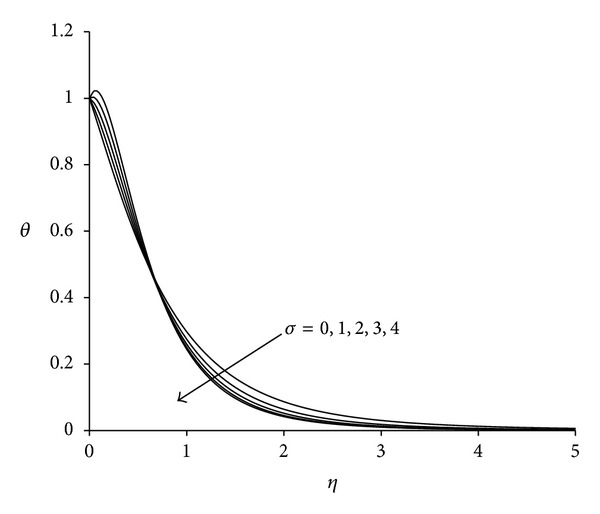
Temperature distribution for different *σ*  (*λ* = 3, *τ* = 1, Ec = 0.75).

**Figure 11:**
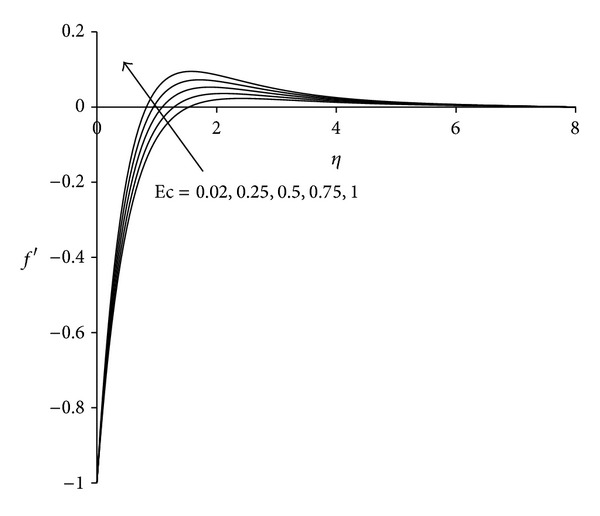
Velocity distribution for different Ec  (*λ* = 3, *τ* = 1, *σ* = 3).

**Figure 12:**
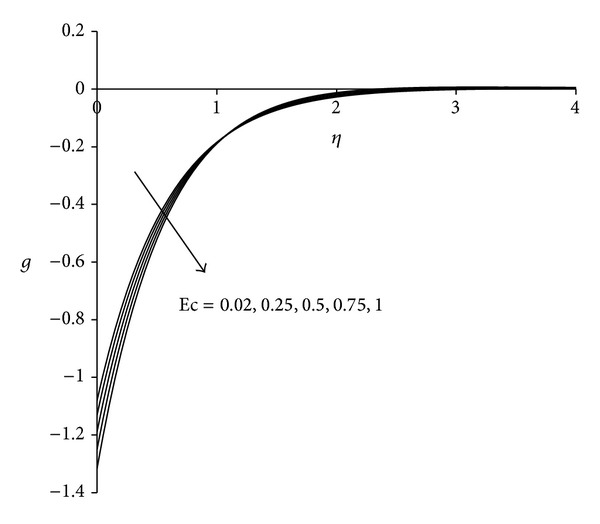
Microrotation distribution for different Ec  (*λ* = 3, *τ* = 1, *σ* = 3).

**Figure 13:**
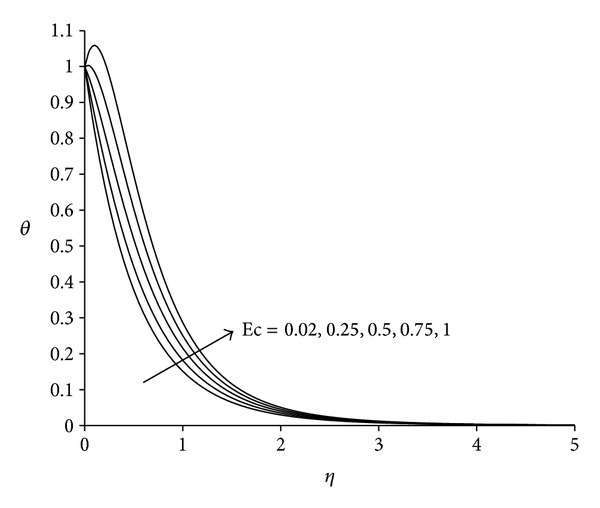
Temperature distribution for different Ec  (*λ* = 3, *τ* = 1, *σ* = 3).

To study the behaviour of velocity, microrotation, and temperature functions, comprehensive numerical computations are carried out for various values of the parameters namely suction parameter *λ*, unsteadiness parameter  *τ*, buoyancy parameter *σ* and Eckert number Ec. The other parameters such as coupling constant parameter *K* and Prandtl number Pr are kept fixed at 2.0 and 0.733 respectively. The results obtained are presented through the graphs as shown in Figures 2–13. The skin friction and the rate of heat transfer have also been computed for these parameters and are tabulated in Tables 3 and 4.

Figures 2–4 display the effect of suction parameter *λ* on the boundary layer flow induced by an unsteady shrinking sheet. Suction is the most suitable force to sustain the flow near the shrinking sheet by confining the generated vorticity inside the boundary layer. It is clear from Figure 2 that the effect of suction parameter *λ* on the velocity is negligible near the sheet whereas away from the sheet velocity decreases with increase in suction parameter. Negative values of velocity close to the sheet indicate the region of reverse flow.  Figure 3 shows that as suction parameter increases the microrotation decreases near the sheet. After covering a small distance from the sheet, the opposite behaviour is observed. The negative values of microrotation show the reverse rotation of microparticles.  Figure 4 illustrates the influence of suction parameter *λ* on the temperature distribution. It is evident from the figure that temperature of the fluid decreases with increase in suction parameter. As suction is applied, thermal boundary layer thickness decreases as a result of which temperature of the fluid in the boundary layer decreases. This observation is in agreement with Bhattacharyya and Layek [25]. Thus, suction parameter can be used effectively for controlling the flow and heat transfer characteristics.

The effect of unsteadiness parameter  *τ* on the velocity, microrotation, and temperature functions is shown in Figures 5–7.  Figure 5 shows that increasing the value of unsteadiness parameter *τ* tends to decrease the velocity in the boundary layer. Near the sheet flow velocity is negative whereas away from the sheet it becomes positive and finally satisfies the free stream boundary condition. Thus close to the sheet the flow is strongly reversed.  Figure 6 demonstrates that, near the sheet the effect of unsteadiness on the microrotation is negligible, but away from the sheet it increases with increase in unsteadiness parameter. After covering a certain distance from the sheet, all profiles converge and finally satisfy the far field boundary condition.  Figure 7 reveals that the temperature decreases with increase in unsteadiness parameter *τ*. Temperature at the sheet is invariant for higher values of unsteadiness parameter. These patterns are consistent with the findings of Ishak et al. [35]. Physically as the value of unsteadiness parameter increases the sheet loses more heat as a result of which temperature of the fluid decreases.

Figures 8–10 display the nature of velocity, microrotation and temperature distributions with buoyancy parameter *σ*. Physically positive values of *σ* are associated with cooling of the sheet (assisting flow), negative values of *σ* indicate heating of the sheet (opposing flow), and *σ* = 0 implies vanishing buoyancy effects. From Figure 8, it can be seen that velocity of the fluid increases with increase in buoyancy parameter. This is due to the reason that an increase in the value of buoyancy parameter leads to an increase in the temperature difference (*T*_*w*_ − *T*_*∞*_). This leads to an increase in the convection currents as a result of which fluid velocity increases and thus, boundary layer thickness decreases. This trend has also been identified by Shit and Haldar [41].  Figure 9 indicates that the microrotation decreases initially with increase in buoyancy parameter. For *η* ≈ 1.15 all profiles intersect and then increases with increases in buoyancy parameter. In all cases microrotation is negative which shows the reverse rotation of microelements. A crossing over point appears in the temperature profile as shown in Figure 10. This point is special where all temperature curves cross each other; that is, the temperature profile shows different behaviour before and after this point. It is observed that as the buoyancy parameter increases, fluid temperature increases up to this point and decreases after this point. Maximum temperature occurs near the sheet corresponding to *σ* = 4. Temperature at the sheet is invariant for lower values of buoyancy parameter.

Figures 11–13 present the distributions of velocity, microrotation and temperature functions with Eckert number Ec. This parameter is called the fluid motion controlling parameter. From Figure 11 it is observed that the velocity increases with increase in Eckert number Ec. Boundary layer thickness will therefore decrease as Eckert number increases. Flow reversal arises near the sheet as testified by the negative values of velocity.  Figure 12 demonstrates that in the vicinity of sheet microrotation decreases with increase in Eckert number. Minimum value of microrotation corresponds to Ec = 1 and this value is −1.32 approximately. After a small distance from the sheet all profiles converge and finally satisfy the far field boundary condition.  Figure 13 reveals that the temperature as well as thermal boundary layer thickness increases with increase in Eckert number Ec. A temperature overshoot near the sheet has been observed for higher values of Eckert number. This is due to the fact that for higher values of Eckert number, there is a significant generation of heat due to viscous dissipation near the sheet, so that the temperature in the region close to the sheet exceeds the temperature of the wall *T*_*w*_.

Table 3 gives the skin friction for different values of *λ*, *τ*, *σ* and Ec. It is evident that the skin friction decreases slightly with a small increase in unsteadiness parameter while it increases with increase in suction parameter, buoyancy parameter, Eckert number and large values of unsteadiness parameters. Physically positive values of the skin friction show that the fluid exerts a drag force on the sheet. It has also been observed that the skin friction is lower in the absence of buoyancy parameter. Thus skin friction can be reduced effectively by assigning lower values to unsteadiness parameter and in the absence of buoyancy parameter. From Table 4 it may be noted that the rate of heat transfer decreases initially with increase in buoyancy parameter, Eckert number small values of suction, and unsteadiness parameters. It is also observed that heat transfer rate increases numerically with increase in suction, unsteadiness parameters, large values of buoyancy parameter, and Eckert number. Positive values of the heat transfer rate indicate that the heat is transferred from the surface of the sheet to the fluid and negative values mean the opposite. Thus effective cooling of the sheet can be achieved by the judicious selection of these parameters.

## 5. Conclusions

The present study has addressed theoretically and numerically the unsteady mixed convection flow and heat transfer of an incompressible micropolar fluid over a porous shrinking sheet in the presence of viscous dissipation. Using similarity transformations the governing time-dependent boundary layer equations are reduced to a set of nonlinear ordinary differential equations. A variational finite element method has been employed to solve the nondimensional momentum, angular momentum, and thermal boundary layer equations, subject to physically realistic boundary conditions. Under limiting cases the numerical results obtained for the flow velocity are compared very well with the exact solution available in the literature. Numerical computations have clearly demonstrated that the drag can be reduced effectively with the judicious selection of unsteadiness parameter, and buoyancy parameter. It has also been found that a fast rate of cooling can be achieved by implementing suction parameter, unsteadiness parameter, higher values of buoyancy parameter and Eckert number. The present study has neglected velocity and thermal slip effects at the sheet. These may be considered in the future. It is hoped that the results obtained from the present work may be useful for different model investigations.

## Figures and Tables

**Table 1:** Convergence of results with the variation of number of elements *n* (*K* = 2, Pr = 0.733,  λ = 3,  τ = 1,  σ = 3,  Ec = 0.75).

*n*	*f* (1.6)	*h* (1.6)	*g* (1.6)	θ(1.6)
20	2.7194	0.0714	−0.1087	0.1857
40	2.6986	0.0524	−0.1198	0.1772
60	2.6955	0.0496	−0.1223	0.1756
80	2.6945	0.0487	−0.1231	0.1751
100	2.6941	0.0483	−0.1236	0.1748
120	2.6939	0.0481	−0.1238	0.1747
140	2.6937	0.0480	−0.1239	0.1746
160	2.6937	0.0479	−0.1240	0.1745
180	2.6936	0.0479	−0.1241	0.1745

**Table 2:** Comparison of the flow velocity *f*′(η) obtained by analytical method [24] and FEM (*K* = 0,  Pr = 0.733,  λ = 3,  τ = 0,  σ = 0,  Ec = 0) in the special case.

η	*f*′(η)	
	[24]	FEM
0	−1	−1
1	−0.07295	−0.07290
2	−0.00532	−0.00531
3	−0.00039	−0.00039
4	−0.00003	−0.00003
5, 6, 7, 8	0.00000	0.00000

**Table 3:** The skin friction coefficient *f*′′(0) for different values of λ, τ, σ, and Ec  (*K* = 2, Pr = 0.733).

$\begin{array}{c} \tau =1,\;\;\sigma =3,\\ \;\;\text{Ec}=0.75 \end{array}$		$\begin{array}{c} \lambda =3,\;\;\sigma =3,\\ \;\;\text{Ec}=0.75 \end{array}$		$\begin{array}{c} \lambda =3,\;\;\tau =1,\\ \;\;\text{Ec}=0.75 \end{array}$		$\begin{array}{c} \lambda =3,\tau =1,\\ \;\;\;\;\sigma =3 \end{array}$	
λ	*f*′′(0)	τ	*f*′′(0)	σ	*f*′′(0)	Ec	*f*′′(0)
3	2.49867	1	2.49867	0	1.31709	0.02	2.15971
3.5	2.58229	2	2.49630	1	1.74698	0.25	2.26044
4	2.70175	3	2.52259	2	2.12938	0.5	2.37579
4.5	2.85110	4	2.57288	3	2.49867	0.75	2.49867
5	3.02241	5	2.63686	4	2.87143	1	2.63105

**Table 4:** The local Nusselt number −θ′(0) for different values of λ, τ, σ, and Ec  (*K* = 2, Pr = 0.733).

$\begin{array}{c} \tau =1,\;\;\sigma =3,\\ \;\;\text{Ec}=0.75 \end{array}$		$\begin{array}{c} \lambda =3,\;\;\sigma =3,\\ \;\;\text{Ec}=0.75 \end{array}$		$\begin{array}{c} \lambda =3,\;\;\tau =1,\\ \;\text{Ec}=0.75 \end{array}$		$\begin{array}{c} \lambda =3,\;\;\tau =1,\\ \;\;\;\;\;\sigma =3 \end{array}$	
λ	−θ′(0)	τ	−θ′(0)	σ	−θ′(0)	Ec	−θ′(0)
3	−0.24924	1	−0.24924	0	0.88895	0.02	1.99596
3.5	0.12914	2	0.14660	1	0.54444	0.25	1.39436
4	0.45990	3	0.46597	2	0.17095	0.5	0.63527
4.5	0.75067	4	0.71556	3	−0.24924	0.75	−0.24924
5	1.01279	5	0.91656	4	−0.73308	1	−1.28127

## Nomenclature

*c*_*p*_: Specific heat at constant pressure
*C*_*f*_: Skin friction coefficient
*f*: Dimensionless velocity
*g*: Dimensionless microrotation
*g*_*e*_: Gravitational acceleration
Gr_*x*_: Local Grashof number
*j*: Microinertia density
*K*: Coupling constant parameter
*N*: Microrotation component
Nu_*x*_: Local Nusselt number
Pr: Prandtl number
*q*: Heat flux
*Re*_*x*_: Local Reynolds number
*S*: Constant characteristic of the fluid
*T*: Temperature of the fluid
*T*_*∞*_: Temperature of the ambient fluid
*t*: Time
*u*: Velocity in the *x*-direction
*U*_*w*_: Velocity of the sheet
*v*: Velocity in the *y*-direction
*V*_*w*_: Velocity at the wall
*w*_*i*_: Weight functions
*x*: Distance along the surface
*y*: Distance normal to the surface.

### Greek Symbols

*γ*: Spin gradient viscosity
*η*: Similarity variables
*μ*: The dynamic viscosity
*ρ*: Density of the fluid
*κ*: Thermal conductivity
*ν*: Kinematic viscosity
*θ*: Dimensionless temperature
*λ*: Suction parameter
*ϕ*: Stream function
*σ*: Buoyancy parameter
*τ*: Unsteadiness parameter
*τ*_*w*_: Wall shear stress.

### Subscripts

*w*: Surface condition
*∞*: Conditions far away from the surface.
